# Influence of Obesity on Short-Term Surgical Outcomes in HFrEF Patients Undergoing CABG: A Retrospective Multicenter Study

**DOI:** 10.3390/biomedicines12020426

**Published:** 2024-02-13

**Authors:** Christian Jörg Rustenbach, Stefan Reichert, Christoph Salewski, Julia Schano, Rafal Berger, Attila Nemeth, Monika Zdanyte, Helene Häberle, Túlio Caldonazo, Ibrahim Saqer, Shekhar Saha, Philipp Schnackenburg, Ilija Djordjevic, Ihor Krasivskyi, Lina María Serna-Higuita, Torsten Doenst, Christian Hagl, Thorsten Wahlers, Christian Schlensak, Rodrigo Sandoval Boburg

**Affiliations:** 1 Department of Thoracic and Cardiovascular Surgery, German Cardiac Competence Center, Eberhard-Karls-University, 72076 Tuebingen, Germany; stefan.reichert@med.uni-tuebingen.de (S.R.); christoph.salewski@med.uni-tuebingen.de (C.S.); julia.schano@med.uni-tuebingen.de (J.S.); rafal.berger@med.uni-tuebingen.de (R.B.); attila.nemeth@med.uni-tuebingen.de (A.N.); christian.schlensak@med.uni-tuebingen.de (C.S.); rodrigo.sandoval-boburg@med.uni-tuebingen.de (R.S.B.); 2 Department of Cardiology, German Cardiac Competence Center, Eberhard-Karls-University, 72076 Tuebingen, Germany; monika.zdanyte@med.uni-tuebingen.de; 3 Department of Anesthesiology and Intensive Care Medicine, Eberhard-Karls-University, 72076 Tuebingen, Germany; helene.haeberle@med.uni-tuebingen.de; 4 Department of Cardiothoracic Surgery, Jena University Hospital, 07747 Jena, Germany; tulio.caldonazo@med.uni-jena.de (T.C.); ibrahim.saqer@med.uni-jena.de (I.S.); doenst@med.uni-jena.de (T.D.); 5 Department of Cardiac Surgery, Ludwig-Maximilians-University, 80539 Munich, Germanyphilipp.schnackenburg@med.uni-muenchen.de (P.S.); christian.hagl@med.uni-muenchen.de (C.H.); 6 Department of Cardiothoracic Surgery, Heart Center, University of Cologne, 50923 Köln, Germany; ilija.djordjevic@uk-koeln.de (I.D.); ihor.krasivskyi@uk-koeln.de (I.K.); thorsten.wahlers@uk-koeln.de (T.W.); 7 Institute for Clinical Epidemiology and Applied Biostatistics, Eberhard-Karls-University, 72076 Tuebingen, Germany; lina.serna-higuita@med.uni-tuebingen.de

**Keywords:** obesity, heart failure, CABG, OPCAB, HFrEF, low EF

## Abstract

**Background**: This retrospective multicenter study investigates the impact of obesity on short-term surgical outcomes in patients with heart failure and reduced ejection fraction (HFrEF) undergoing coronary artery bypass grafting (CABG). Given the rising global prevalence of obesity and its known cardiovascular implications, understanding its specific effects in high-risk groups like HFrEF patients is crucial. **Methods**: The study analyzed data from 574 patients undergoing CABG across four German university hospitals from 2017 to 2023. Patients were stratified into ‘normal weight’ (*n* = 163) and ‘obese’ (*n* = 158) categories based on BMI (WHO classification). Data on demographics, clinical measurements, health status, cardiac history, intraoperative management, postoperative outcomes, and laboratory insights were collected and analyzed using Chi-square, ANOVA, Kruskal–Wallis, and binary logistic regression. **Results**: Key findings are a significant higher mortality rate (6.96% vs. 3.68%, *p* = 0.049) and younger age in obese patients (mean age 65.84 vs. 69.15 years, *p* = 0.003). Gender distribution showed no significant difference. Clinical assessment scores like EuroScore II and STS Score indicated no differences. Paradoxically, the preoperative left ventricular ejection fraction (LVEF) was higher in the obese group (32.04% vs. 30.34%, *p* = 0.026). The prevalence of hypertension, COPD, hyperlipidemia, and other comorbidities did not significantly differ. Intraoperatively, obese patients required more packed red blood cells (*p* = 0.026), indicating a greater need for transfusion. Postoperatively, the obese group experienced longer hospital stays (median 14 vs. 13 days, *p* = 0.041) and higher ventilation times (median 16 vs. 13 h, *p* = 0.049). The incidence of acute kidney injury (AKI) (17.72% vs. 9.20%, *p* = 0.048) and delirium (*p* = 0.016) was significantly higher, while, for diabetes prevalence, there was an indicating a trend towards significance (*p* = 0.051) in the obesity group, while other complications like sepsis, and the need for ECLS were similar across groups. **Conclusions**: The study reveals that obesity significantly worsens short-term outcomes in HFrEF patients undergoing CABG, increasing risks like mortality, kidney insufficiency, and postoperative delirium. These findings highlight the urgent need for personalized care, from surgical planning to postoperative strategies, to improve outcomes for this high-risk group, urging further tailored research.

## 1. Introduction

Coronary Artery Bypass Grafting (CABG) stands as a pivotal intervention in cardiac surgery, particularly for patients grappling with Heart Failure with reduced Ejection Fraction (HFrEF). The complex dynamics of managing HFrEF, a condition marked by significantly impaired cardiac output, necessitate an in-depth exploration of various preoperative risk factors such as obesity, which is increasingly prevalent globally and has a well-documented impact on cardiovascular health [1,2].

Recent research underscores the nuanced role of obesity in CABG outcomes [3,4]. A study revealed that obesity does not significantly escalate in-hospital mortality or prolong ICU and hospital stays in CABG patients, challenging earlier assumptions about the risks associated with obesity in cardiac surgeries [5,6]. In the context of grafting techniques, research has shown that outcomes like postoperative mediastinitis in obese patients may not be significantly impacted by the choice of grafting technique, further complicating the understanding of obesity’s role in surgical outcomes [7,8].

The specific impact of obesity on short-term outcomes in HFrEF patients undergoing CABG remains unexplored. This particular patient group presents a unique intersection of challenges due to their compromised cardiac function, which could interact differently with obesity, potentially affecting surgical outcomes in ways not yet fully understood.

This multicenter, retrospective study aims to address this gap. By focusing on the short-term effects of obesity in HFrEF patients undergoing CABG, it seeks to unveil critical insights into patient care in this subgroup. The study’s findings could significantly contribute to the evolving field of personalized medicine in cardiac surgery. With the ever-increasing prevalence of obesity, understanding its specific implications for high-risk groups such as HFrEF patients is becoming increasingly crucial. This research aims not only to enrich the existing body of knowledge but also to inform clinical practices, potentially leading to more refined surgical planning and improved outcomes for this vulnerable patient cohort.

## 2. Materials and Methods

### 2.1. Study Design and Patient Population

We conducted a multicenter, retrospective study on patients with Heart Failure with reduced Ejection Fraction (HFrEF) who underwent Coronary Artery Bypass Grafting (CABG) from 2017 to 2023. The data were gathered from four university hospitals in Germany. Key data were collected encompassing demographics, clinical measurements, health status, cardiac history, intraoperative management, and postoperative outcomes. This included age, gender, BMI, EuroScore II, STS Score, LVEF, comorbidities (e.g., diabetes, hypertension, COPD), preoperative cardiac rhythm, and history of myocardial infarction or percutaneous coronary interventions (PCIs). Adult patients (aged 18 and above) undergoing CABG with a preoperative diagnosis of HFrEF (LVEF < 40%) were included. Exclusions were patients with incomplete data, emergency CABG cases, or significant valvular diseases requiring concurrent surgery. Patients were classified into ‘Normal weight’ (BMI of 18.5–24.9) and ‘Obesity’ categories, the latter combining Obesity Class I (BMI of 30–34.9), Class II (35–39.9), and Class III (≥40), following WHO guidelines. This categorization was pivotal in assessing the impact of obesity on surgical outcomes.

Additionally, we conducted a subgroup analysis of postoperative outcomes between the Normal Weight and Overweight and the Overweight and Obesity groups to more clearly and robustly delineate the relationship between BMI and outcomes, as detailed in the Supplementary Materials (Tables S1 and S2).

### 2.2. Description of Surgical Procedures

The detailed description of the surgical CABG procedures (ONCAB and OPCAB), including specific techniques and protocols, is extensively elaborated in our prior study. For an in-depth understanding of these surgical methodologies, readers are encouraged to refer to that publication [9].

### 2.3. Statistical Analysis

Our statistical approach was modeled after our previous research, where we observed varying rates of a composite outcome (in-hospital mortality, prolonged ventilation, renal failure) across different BMI categories in patients with heart failure and reduced ejection fraction (HFrEF) undergoing coronary artery bypass grafting (CABG). To detect significant differences in outcomes between these two groups, we employed various statistical tools. Baseline, intraoperative, and postoperative clinical characteristics were compared using Chi-square tests for categorical variables and Analysis of Variance (ANOVA) for continuous variables. For non-parametric data, the Kruskal–Wallis test was applied.

In cases of missing data, patients with incomplete data were either excluded from the analysis or a sensitivity analysis was conducted to account for potential biases. The initial fully adjusted regression model included all predictor variables with an unadjusted association of at least *p* ≤ 0.20 with the composite outcome. To prevent collinearity, we evaluated the relationships among novel measures using Pearson and Spearman correlations.

To adjust for potential confounders and to assess the impact of obesity on surgical outcomes, we conducted a binary logistic regression analysis. This included factors like preoperative health status, intraoperative resource utilization, and postoperative complications.

All analyses were conducted using SPSS (IBM^®^ SPSS Statistics, Version 28.0, IBM Corp., Armonk, NY, USA) and Microsoft Excel (Microsoft Corporation, 2019) for data organization. This comprehensive approach ensured robust and reliable insights into the impact of obesity on surgical outcomes in HFrEF patients undergoing CABG.

## 3. Results

### 3.1. Preoperative Demographic and Clinical Profile

Our retrospective multicenter study scrutinized a cohort of 574 patients with heart failure and reduced ejection fraction (HFrEF) undergoing coronary artery bypass grafting (CABG). This cohort was stratified into two distinct BMI categories: ‘Normal weight’ encompassing 163 patients (28.4%) and ‘Obesity’ including 158 patients (27.5%). The demographical divide revealed a statistically significant younger mean age in the obese group (65.84 ± 10.00 years) compared to their normal weight counterparts (69.15 ± 9.85 years, *p* = 0.003, ANOVA). Despite the significant age disparity, gender distribution across the groups did not demonstrate statistical significance, with males representing the majority (87.80%).

Clinical assessment scores such as EuroScore II and STS Score, which prognosticate surgical risk, showed no notable differences between the groups. However, the preoperative left ventricular ejection fraction (LVEF) was marginally higher in the obesity group (*p* = 0.026, ANOVA), indicating a subtle but measurable variance in cardiac function related to body weight.

We evaluated the prevalence of key comorbid conditions, including diabetes (both oral antidiabetic and insulin-dependent types), smoking history, hypertension, COPD, hyperlipidemia, preoperative stroke, carotid stenosis, peripheral artery disease (PAD), and renal insufficiency, to assess their impact on the patients’ overall health status and surgical risk. Our analysis revealed no significant differences in these comorbidities between the normal weight and obese groups. Additionally, pre-operative cardiac conditions, including sinus rhythm versus atrial fibrillation and history of myocardial infarction (both NSTEMI and STEMI), as well as previous percutaneous coronary interventions (PCIs), were compared, showing no significant differences between the groups. The comprehensive data on clinical measurements, health status, and cardiac history provide a contextual backdrop for interpreting the surgical outcomes and postoperative recovery within this cohort. These facets are crucial for devising personalized care strategies and can be found in detail in Table 1.

### 3.2. Intraoperative Management

**Figure 1:**
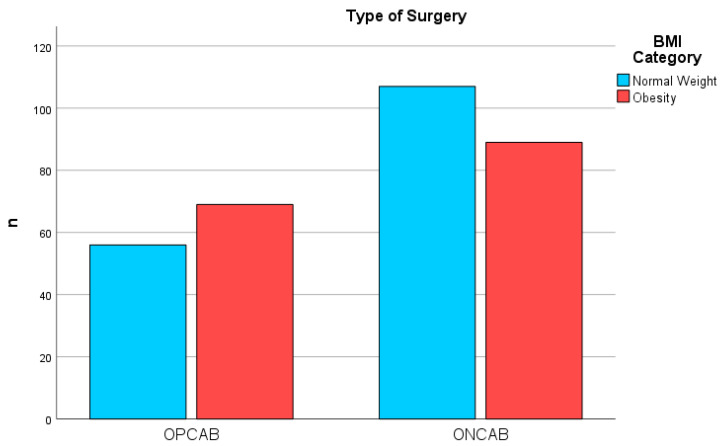
Surgical Methods by BMI Category. Distribution of OPCAB and ONCAB Surgical Procedures Among Patients Classified by BMI Category, showing comparative frequencies between normal weight and obesity groups.

Surgical techniques were distributed evenly across the BMI categories, with no significant preference for off-pump (OPCAB) versus on-pump (ONCAB) coronary artery bypass methods (Table 2 and Figure 1). When delving into the intraoperative resource utilization, obese patients required a higher mean of packed red blood cells (PRB), indicating a more considerable intraoperative transfusion need (*p* = 0.002, ANOVA). In contrast, the usage of platelets (PLTP) and fresh frozen plasma (FFP) was comparable between the groups, suggesting that the higher transfusion rates in the obese group might be attributed to factors other than bleeding diathesis. These parameters are detailed in Table 3.

### 3.3. Postoperative Outcomes and Laboratory Insights

**Figure 2:**
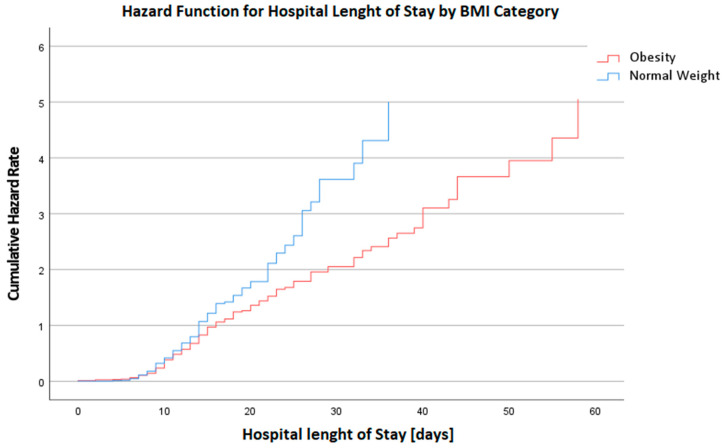
Hazard Function for Hospital Length of Stay in days by BMI Category. This graph depicts the cumulative hazard rates for the duration of hospital stay, categorized by BMI. A higher hazard rate is observed in the BMI Category 4 group, indicating an increased risk for prolonged hospital stays or complications among these patients.

The postoperative phase highlighted disparities primarily in the length of stay at the hospital (LOS@Hospital), with the median duration slightly elongated for the obese group (14 days vs. 13 days for the normal weight group, *p* = 0.041, MW), visualized in Figure 2. Ventilation time (Vent) post-surgery also trended higher in the obese group, although marginally (16 h vs. 13 h, *p* = 0.049, MW). These findings suggest that obesity may contribute to a more protracted and complicated postoperative recovery trajectory. In the postoperative period after chest closure, the requirement for vasopressor and inotropic support was scrutinized. The median requirement for epinephrine was zero across both groups, but the interquartile range extended up to 0.05 in the normal weight group and 0.02 in the obese group, indicating a higher upper range of epinephrine use in the normal weight patients (*p* = 0.048, MW). For norepinephrine, both groups had a median usage of 0.10, with the normal weight group using a slightly broader range of dosages (0.06–0.17) compared to the obese group (0–0.10), though this difference did not reach statistical significance (*p* = 0.120, MW).

Preoperative laboratory results illuminated that obese patients exhibited significantly higher hemoglobin levels (*p* = 0.019, TT), whereas other key markers such as creatinine, GFR, cardiac enzymes (CK, CK-MB, troponin I (HS)), and lactate maintained homogeneity across the BMI spectrum, signifying a nuanced impact of obesity on the biochemical milieu. These findings are elaborated in Table 3.

### 3.4. Complications and Morbidity

Noteworthy in the postoperative complications was the increased incidence of acute kidney injury (AKI) in the obese group (*p* = 0.048, Chi2). Delirium occurrence was significantly higher in the obese cohort (*p* = 0.016, Chi²), which warrants attention for perioperative care strategies. The mortality showed a significant higher risk in the obese group, underscoring the potential amplified risk that obesity might pose in the backdrop of cardiac surgery (*p* = 0.049, Chi²), although the nature of this association is unclear.

Infection-related complications, represented by the incidence of sepsis, were not significantly different between the groups, indicating that obesity per se did not escalate the infectious risk in this context. The rate of resternotomy and extracorporeal life support (ECLS) usage was also similar, suggesting that immediate surgical outcomes were not predominantly influenced by obesity status.

These findings are elaborated in Table 4.

## 4. Discussion

The influence of obesity on the outcomes of coronary artery bypass grafting (CABG) in patients with heart failure and reduced ejection fraction (HFrEF) is a subject of significant clinical interest. Our multicenter study sheds light on the perioperative and postoperative nuances that accompany these complex surgeries in the context of varying body mass indices (BMI). To date, there is a lack of studies on obesity in CABG surgery in this special and vulnerable patient cohort.

### 4.1. Preoperative Demographic and Clinical Profile

Our study’s demographic findings are consistent with literature that associates obesity with younger age at CABG surgery [10]. This demographic trend poses a particular challenge, as these patients may endure longer cumulative years of comorbidity and potentially require repeat interventions.

Interestingly, no significant differences in gender distribution were found in our study, although the proportion of men was higher, which contrasts with the current literature suggesting a higher prevalence of obesity in men with cardiovascular disease [11]. The EuroSCORE II and STS scores, which predict cardiac surgery risks, showed no significant differences between groups, reflecting a complex risk profile where obesity is one of many factors. In addition, the EuroSCORE II model does not incorporate BMI as a risk factor for perioperative mortality stratification [12].

One intriguing observation was the marginally higher preoperative left ventricular ejection fraction (LVEF) in the obese group, a finding that contradicts the commonly held belief that obesity is uniformly detrimental to cardiac function. This could suggest a potential ‘obesity paradox’, where certain aspects of cardiac function may be preserved or even enhanced in obese individuals, as discussed in the literature [13,14].

In our study, the borderline *p*-value of 0.051 regarding the prevalence of diabetes (both oral antidiabetic and insulin-dependent) in the normal weight and obese groups invites a nuanced discussion. This *p*-value, while just exceeding the traditional threshold for statistical significance, indicates a trend that warrants attention. This is particularly relevant given the established links between obesity and diabetes in the wider literature. The relationship between obesity and diabetes has been well-documented, with obesity recognized as a significant risk factor for the outcome in cardiac surgery [4,15]. However, our findings indicate that within the context of patients undergoing CABG, this relationship might not be as pronounced. This could suggest that other factors, such as the severity of cardiac disease, might play a more influential role in the prevalence of diabetes in this specific patient population.

For clinicians, the marginal *p*-value highlights the importance of considering factors beyond BMI when evaluating the risk and presence of diabetes in cardiac surgery patients. It suggests that comprehensive patient assessments, which include but are not limited to BMI, are crucial for accurate risk stratification and management. The borderline *p*-value in our study could serve as an impetus for future research.

### 4.2. Intraoperative Management

In our study, contrary to expectations, we observed higher mean requirements for packed red blood cells in obese patients undergoing surgery. This finding raises intriguing questions about the interplay between obesity, hemostasis, and bleeding risk. While some studies have suggested that obesity may confer a lower risk of bleeding complications [16,17], others have presented conflicting evidence [18]. This suggests that any presumed protection against bleeding in obesity, attributed to an apparently efficient hemostatic system, may be offset by other factors. Furthermore, it remains presently unclear whether the prothrombotic state associated with obesity is sufficient to protect against clinically relevant bleeding, as supported by recent reviews [19].

Adding to the complexity of this issue, a large registry-based study investigating the impact of obesity on postoperative outcomes, with a focus on bleeding and transfusion requirements, found that patients with high BMI may actually exhibit protection against postoperative bleeding following cardiac surgery, possibly due to an inherent hypercoagulable state [16]. It is worth noting that preoperative hypertension is a well-known major risk factor for postoperative bleeding [20]. In addition to these factors, several variables, including the size of the surgical area, the necessity for arbitrary protamine reversal due to uncertain plasma heparin concentration, and inadequate warming, may exacerbate bleeding in high-BMI patients [21]. It is important to acknowledge that our study did not include an analysis of coagulation factors or their functionality, which leaves us with an incomplete understanding of why we detected an increased transfusion requirement in obese patients. While the clinical implications are multifaceted, our findings emphasize the importance of tailored intraoperative management for obese patients. This may entail the implementation of more aggressive strategies for bleeding control and transfusion in this specific patient population. Further studies in this area are warranted to elucidate the intricate relationship between obesity, coagulation, and bleeding risk.

### 4.3. Postoperative Outcomes and Laboratory Insights

The prolonged hospital length of stay and increased ventilation time in the obese group of HFrEF patients are consistent with the literature, which often reports extended recovery periods for obese patients [22,23] following CABG. This could be attributed to a higher burden of comorbidities, increased surgical complexity, and the challenges in postoperative care, including ventilation management.

The preoperative laboratory values, particularly the higher hemoglobin levels in the obese group, present a counterintuitive aspect often not discussed in existing studies [24]. While it is widely acknowledged that state that could affect various laboratory parameters, our findings suggest that the relationship between obesity and preoperative biochemical status may be more nuanced.

### 4.4. Complications and Morbidity

The increased incidence of postoperative complications, such as AKI and delirium, in the obese group is a critical concern. While the increased incidence of AKI in our obese group aligns with the literature [25], the higher risk of delirium contrasts with some studies [26]. These complications have significant implications for patient outcomes and healthcare resources, emphasizing the need for heightened vigilance and possibly preventive strategies in the management of obese patients in this special cohort.

The trend toward higher mortality in the obese group echoes the sentiments of several studies that implicate obesity as a factor in increased short-term mortality following CABG. However, the ‘obesity paradox’—where obesity, in certain contexts, appears to confer a survival advantage after cardiac events—remains a contentious topic, with some studies suggesting a protective effect of increased BMI in the postoperative period [22,27,28,29], but is contradicted in other studies [30,31].

## 5. Conclusions

Obesity significantly worsens short-term surgical outcomes in HFrEF patients undergoing isolated CABG surgery. Firstly, despite their younger age, these patients are at a significantly higher risk for mortality, albeit with a slightly higher LVEF. Secondly, they exhibit a significant higher incidence of acute kidney insufficiency and postoperative delirium, as well as suffer from prolonged ventilation times and hospital stays. Thirdly, and paradoxically, they experience increased intraoperative transfusion requirements. These observations underscore the need for tailored surgical and pre, and postoperative strategies to mitigate the adverse effects of obesity, highlighting the critical need for personalized care in this high-risk patient population. Prospective studies with larger cohorts and extended follow-up periods are critically needed to understand the long-term impact of obesity on CABG outcomes.

## Figures and Tables

**Table 1:** Preoperative Demographic Data and Clinical Measurements by BMI Categories.

Variable	Total Cohort	Normal Weight	Obesity	*p* Value
	(*n* = 574)	(*n* = 163) 28.4%	(*n* = 158) 27.5%	
				
**Demographic Data**				
Age, mean (±SD)	67.82 (±9.89)	69.15 (±9.85)	65.84 (±10.00)	0.003 ^ANOVA^
Gender				
Male *n* (%)	504 (87.80%)	144 (88.34%)	134 (84.81%)	0.353 ^Chi²^
Female *n* (%)	70 (12.19%)	19 (11.65%)	24 (15.19%)	
BMI, mean (±SD)	27.91 (±5.01)	23.03 (±1.50)	34.15 (±4.04)	<0.001 ^ANOVA^
				
**Clinical Measurements**				
EuroScore II, mean (±SD)	5.36 (±5.08)	4.99 (±4.32)	5.23 (±4.87)	0.648 ^ANOVA^
STS Score, mean (±SD)	2.65 (±2.31)	2.56 (±2.06)	2.60 (±2.43)	0.860 ^ANOVA^
LVEF preop, mean (±SD)	31.51 (±6.97)	30.34 (±7.06)	32.04 (±6.46)	0.026 ^ANOVA^
				
**Health Status**				
Diabetes				
OAD *n* (%)	134 (23.3%)	37 (22.69%)	39 (24.68%)	0.051 ^Chi²^
Insulin dependent *n* (%)	138 (24.0%)	33 (19.64%)	48 (27.22%)	
Smoking history				
Former *n* (%)	157 (27.4%)	38 (23.31%)	52 (32.91%)	0.131 ^Chi²^
Active *n* (%)	117 (20.4%)	31 (19.13%)	29 (17.94%)	
Hypertension, *n* (%)	534 (93.03%)	151 (92.6%)	151 (95.56%)	0.266 ^Chi2^
COPD, *n* (%)	111 (19.3%)	33 (20.25%)	36 (22.78%)	0.580 ^Chi2^
Hyperlipidemia, *n* (%)	484 (84.6%)	138 (84.66%)	136 (86.08%)	0.617 ^Chi²^
Stroke preOP, *n* (%)	59 (10.27%)	15 (9.20%)	16 (10.12%)	0.779 ^Chi²^
Carotid Stenosis, *n* (%)	95 (10.27%)	34 (9.20%)	27 (17.09%)	0.389 ^Chi²^
PAD, *n* (%)	131 (22.82%)	33 (20.46%)	35 (22.15%)	0.676 ^Chi²^
Renal insufficiency, *n* (%)	172 (29.97%)	44 (26.99%)	45 (28.48%)	0.845 ^Chi²^
				
**Cardiac History**				
Rhythm, pre-operative				
SR, *n* (%)	378 (65.85%)	116 (71.17%)	101 (63.92%)	0.296 ^Chi²^
Afib, *n* (%)	192 (33.45%)	47 (28.83%)	55 (34.81%)	
NSTEMI, *n* (%)	188 (32.75%)	52 (31.90%)	53 (33.54%)	0.754 ^Chi2^
STEMI, *n* (%)	86 (14.98%)	25 (15.34%)	19 (12.03%)	0.388 ^Chi2^
Previous PCI, *n* (%)	206 (35.89%)	54 (33.13%)	65 (41.14%)	0.126 ^Chi2^
Classification of surgery				
Elective, *n* (%)	314 (54.7%)	86 (52.76%)	90 (56.96%)	0.695 ^Chi²^
Urgent, *n* (%)	173 (30.1%)	50 (30.67%)	42 (26.58%)	
Emergent, *n* (%)	87 (15.2%)	27 (16.56%)	26 (16.46%)	

ANOVA = Anaylsis of Variance.

**Table 2:** Distribution of Surgical Methods by BMI Categories.

BMI Category	Normal Weight	Obesity	Total (*n*, %)
OPCAB (*n*, %)	56 (34.35%)	69 (43.67%)	125 (51.0%)
ONCAB (*n*, %)	107 (65.64%)	89 (56.33%)	196 (48.9%)
Total, *n* (%) by BMI Cateory	163 (50.78%)	158 (49.22%)	321 (100%)

**Table 3:** Intra- and postoperative parameters by BMI Categories.

Variable	Total Cohort	Normal Weight	Obesity	*p* Value
	(*n* = 574)	(*n* = 163)	(*n* = 158)	
		28.4%	27.5%	
**Intraoperative Requirement for Transfusion**				
PRB, mean (±SD)	0.48 (±1.08)	0.35 (±0.88)	0.72 (±1.29)	0.002 ^ANOVA^
PLTP, mean (±SD)	0.34 (±0.69)	0.44 (±0.79)	0.28 (±0.61)	0.142 ^ANOVA^
FFP, mean (±SD)	0.27 (±1.07)	0.34 (±1.22)	0.30 (±1.15)	0.602 ^ANOVA^
**Postoperative (after chest closure) Vasopressor and Inotropic requirements**				
Epinephrine, median (IQR)	0 (0–0.04)	0 (0–0.05)	0 (0–0.02)	0.048 ^MW^
Norepinephrine, median (IQR)	0.10 (0.06–0.16)	0.11 (0.06–0.17)	0.1 (0–0.10)	0.120 ^MW^
**Postoperative Parameters and Complications**				
LOS@Hospital (d), median (IQR)	13 (9–18)	13 (9–16.5)	14 (10–21)	0.041 ^MW^
LOS@ICU (h), median (IQR)	92 (42–144)	87 (46–143)	95 (42–165)	0.163 ^MW^
Vent (h), median (IQR)	13 (8–23)	13 (8–28)	16 (8–31)	0.049 ^MW^
**Laboratory values pre OP**				
Hb (g/dl), mean (±SD)	13.40 (±1.95)	13.06 (±1.91)	13.56 (±1.89)	0.019 ^TT^
Creatinine (mg/dl), mean (±SD)	2.62 (±11.07)	2.23 (±9.52)	2.26 (±9.74)	0.976 ^TT^
GFR (ml/min/BSA), mean (±SD)	76.35 (±25.68)	77.37 (±27.11)	75.77 (±24.29)	0.580 ^TT^
CK (U/L), mean (±SD)	228.98 (±618.20)	204.65 (±538.83)	285.14 (±839.72)	0.363 ^TT^
CK-MB (U/L), mean (±SD)	48.41 (±79.35)	41.44 (±62.57)	45.36 (±63.67)	0.724 ^TT^
Trop I (HS) (ng/L), mean (±SD)	1033.61 (±3770.32)	811.72 (±3171.76)	1216.98 (±3799.54)	0.416 ^TT^
Lactate (mmol/L), mean (±SD)	0.97 (±0.57)	1.03 (±0.65)	0.96 (±0.49)	0.316 ^TT^

ANOVA = Anaylsis of Variance. MW = Mann-Whitney U Test. TT = T-Test.

**Table 4:** Binary logistic regression of postoperative complications.

Variable	Total Cohort	Normal Weight	Obesity	*p* Value	OR	95% CI	*p* Value
	(*n* = 574)	(*n* = 163)	(*n* = 158)				
		28.4%	27.5%				
Resuscitation, *n* (%)	20 (3.48%)	8 (4.91%)	4 (2.53%)	0.267 ^Chi²^	0.671	0.29–2.36	0.999 ^Wald^
Resternotomy, *n* (%)	36 (6.27%)	12 (7.36%)	11 (6.96%)	0.902 ^Chi²^	0.956	0.22–4.15	0.952 ^Wald^
ECLS, *n* (%)	46 (85.18%)	14 (8.58%)	17 (10.75%)	0.348 ^Chi²^	1.219	0.37–4.06	0.747 ^Wald^
AKI, *n* (%)	74 (12.89%)	15 (9.20%)	28 (17.72%)	0.048 ^Chi²^	2.424	0.14–1.28	0.129 ^Wald^
Dialysis, *n* (%)	66 (11.49%)	16 (9.82%)	21 (13.29%)	0.452 ^Chi²^	1.124	0.16–3.18	0.669 ^Wald^
OPCAB, *n* (%)	233 (40.59%)	56 (34.36%)	69 (43.67%)	0.087 ^Chi²^			
ONCAB, *n* (%)	339 (59.06%)	107 (65.64%)	89 (56.33%)				
Stroke, *n* (%)	19 (3.31%)	2 (1.23%)	5 (3.29%)	0.174 ^Fish^	0.902	0.12–2.19	0.399 ^Wald^
Delirium, *n* (%)	98 (17.07%)	22 (13.49%)	41 (26.58%)	0.016 ^Chi²^	2.523	0.26–1.05	0.068 ^Wald^
Mortality, *n* (%)	30 (5.22%)	6 (3.68%)	11 (6.96%)	0.049 ^Chi²^	2.672	0.10–4.45	0.681 ^Wald^
Sepsis, *n* (%)	40 (6.96%)	11 (6.75%)	10 (6.33%)	0.837 ^Chi²^	4.591	0.99–21.12	0.050 ^Wald^

Wald = Wald Test. Fish = Fisher’s Exact Test.

## Data Availability

Data are contained within the article and the foundational research data can be made available upon request in compliance with the EU’s General Data Protection Regulation (GDPR). To ensure compliance, we will seek legal counsel in this matter.

## Supplementary Materials

The following supporting information can be downloaded at: https://www.mdpi.com/article/10.3390/biomedicines12020426/s1, Table S1: Binary logistic regression of postoperative complications in Normal weight (BMI > 18.5–24.9) vs. Overweight (BMI 25–29.9); Table S2: Binary logistic regression of postoperative complications in Overweight (BMI 25–29.9) vs. Obesity (BMI > 35).
