# Supplementary material for: Influence of Obesity on Short-Term Surgical Outcomes in HFrEF Patients Undergoing CABG: A Retrospective Multicenter Study
Source: Biomedicines. 2024 Feb 13;12(2):426. doi: 10.3390/biomedicines12020426 (PMC10887226; doi:10.3390/biomedicines12020426)
Supplement: Supplementary file 1 [file biomedicines-12-00426-s001.zip › biomedicines-2859334-supplementary.pdf]

Supplementary Materials

Table S1: Binary logistic regression of postoperative complications in Normal weight (BMI > 18,5 – 24,9) vs. Overweight (BMI 25 – 29,9).

Variable	Total cohort (n = 574) 100%	Normal weight (n = 163) 28,4%	Overweight (n = 245) 42,68%	p value	OR	95% CI	p value
Resuscitation, n (%)	20 (3,48%)	8 (4,91%)	8 (3,29%)	0,412[Chi²]	0,224	0,04 – 1,26	0,090[Wald]
Resternotomy, n (%)	36 (6,27%)	12 (7,36%)	14 (5,76%)	0,518[Chi²]	0,997	0,27 – 3,62	0,996[Wald]
ECLS, n (%)	46 (85,18%)	14 (8,58%)	17 (6,10%)	0,110[Chi²]	0,609	0,23 – 1,60	0,313[Wald]
AKI, n (%)	74 (12.89%)	15 (9,20%)	31 (15,05%)	0,118[Chi²]	0,671	0,27 – 1,63	0,379[Wald]
Dialysis, n (%)	66 (11,49%)	16 (9,82%)	29 (11,98%)	0,770[Chi²]	1,23	0,39 – 3,89	0,722[Wald]
OPCAB, n (%)	233 (40,59%)	56 (34,36%)	101 (41,22%)	0,470[Chi²]			
ONCAB, n (%)	339 (59,06%)	107 (65,64%)	136 (55,51%)				
Stroke, n (%)	19 (3,31%)	2 (1,23%)	10 (4,13%)	0,101[Chi²]	1,603	0,39 – 6,59	0,514[Wald]
Delirium, n (%)	98 (17,07%)	22 (13,49%)	35 (14,29%)	0,841[Chi²]	0,489	0,26 – 0,95	0,033[Wald]
Mortality, n (%)	30 (5,22%)	6 (3,68%)	15 (6,12%)	0,101[Chi²]	2,028	0,44 – 9,22	0,418[Wald]
Sepsis, n (%)	40 (6,96%)	11 (6,75%)	19 (7,76%)	0,703[Chi²]	1,578	0,52 – 4,76	<0,001[Wald]

Table S2: Binary logistic regression of postoperative complications in Overweight (BMI 25 – 29,9) vs. Obesity (BMI > 35)

Variable	Total cohort (n = 574) 100%	Overweight (n = 245) 42,68%	Obesity (n = 158) 27,5%	p value	OR	95% CI	p value
Resuscitation, n (%)	20 (3,48%)	8 (3,29%)	4 (2,53%)	0,670$^{Chi^2}$	1,121	0,11 – 1,91	0,999^{Wald}
Resternotomy, n (%)	36 (6,27%)	14 (5,76%)	11 (6,96%)	0,615$^{Chi^2}$	1,043	0,27 – 4,09	0,951^{Wald}
ECLS, n (%)	46 (85,18%)	17 (6,10%)	17 (10,75%)	0,285$^{Chi^2}$	0,712	0,35 – 3,27	0,912^{Wald}
AKI, n (%)	74 (12.89%)	31 (15,05%)	28 (17,72%)	0,552$^{Chi^2}$	1,137	0,27 – 1,98	0,531^{Wald}
Dialysis, n (%)	66 (11,49%)	29 (11,98%)	21 (13,29%)	0,595$^{Chi^2}$	1,043	0,32 – 6,11	0,643^{Wald}
OPCAB, n (%)	233 (40,59%)	101 (41,22%)	69 (43,67%)	0,639$^{Chi^2}$			
ONCAB, n (%)	339 (59,06%)	136 (55,51%)	89 (56,33%)				
Stroke, n (%)	19 (3,31%)	10 (4,13%)	5 (3,29%)	0,581$^{Chi^2}$	0,527	0,47 – 4,11	0,999^{Wald}
Delirium, n (%)	98 (17,07%)	35 (14,29%)	41 (26,58%)	0,139$^{Chi^2}$	1,903	0,61 – 2,60	0,527^{Wald}
Mortality, n (%)	30 (5,22%)	15 (6,12%)	11 (6,96%)	0,855$^{Chi^2}$	1,033	0,53 – 2,09	0,241^{Wald}
Sepsis, n (%)	40 (6,96%)	19 (7,76%)	10 (6,33%)	0,600$^{Chi^2}$	0,663	0,52 – 5,87	0,368^{Wald}
